# Supplementary material for: Compare the performance of the models in art classification
Source: PLoS One. 2021 Mar 12;16(3):e0248414. doi: 10.1371/journal.pone.0248414 (PMC7954342; doi:10.1371/journal.pone.0248414)
Supplement: Attachment — Submitted filename: Response to Reviewers.pdf [file pone.0248414.s001.pdf]

Journal: PLOS ONE

Paper Title: Compare the performance of the models in art classification

Manuscript ID: PONE-D-20-34898

Date: February 17, 2021

Response to Reviewer's Comments

The authors would like to thank the editor, academic editor and reviewers for their valuable comments and suggestions to improve the quality of our manuscript. The manuscript has been revised according to the comments. The detailed modifications of the manuscript and the answers to the questions are listed as follows, where **"C"** refers to the word "Comment", while **"R"** refers to "Response".

To Academic Editor

C1: Please ensure that your manuscript meets PLOS ONE's style requirements, including those for file naming. The PLOS ONE style templates can be found at link1 and link2.

R1: We used PLOS template to organize the style of our manuscript, and after a careful check with the PLOS ONE style requirements, we confirmed that our manuscript meets the requirements, including those for file naming. If there are still some mistakes, please contact us. Thank you.

C2: Please amend either the title on the online submission form (via Edit Submission) or the title in the manuscript so that they are identical.

R2: We apologize for our negligence in using different titles on the online submission form and the manuscript. Our title is "Compare the performance of the models in art classification" and we have changed the title in the manuscript.

C3: We note that Figure(s) 1, 2, 3, 6 and 7 in your submission contain copyrighted images. All PLOS content is published under the Creative Commons Attribution License (CC BY 4.0), which means that the manuscript, images, and Supporting Information files will be freely available online, and any third party is permitted to access, download, copy, distribute, and use these materials in any way, even commercially, with proper attribution. For more information, see our copyright guidelines:link. We require you to either (1) present written permission from the copyright holder to publish these figures specifically under the CC BY 4.0 license, or (2) remove the figures from your submission.

R3: We are sorry for our negligence in properly using copyrighted images. We have tried our best to solve the copyright problem. When searching for copyright law of the United States, we found the information below: All works published in the United States before 1924 are in the public domain. Works published after 1923 but before 1978 are protected for 95 years from the date of publication. If the work was created but not published before 1978, the copyright lasts for the life of the author plus 70 years. The Key, 1946 by Jackson Pollock is a copyrighted image, and we have deleted it. We have deleted Fig 1 and Fig 2 because I am not sure whether some paintings have copyrights,

and they had no impact on the structure of the article. The paintings by Maurice Brazil Prendergast (October 10, 1858 – February 1, 1924), Paul Cézanne (19 January 1839 – 22 October 1906) and Vincent Willem van Gogh (30 March 1853 – 29 July 1890) are all in the public domain. We confirm that all paintings in Fig 1 (new) do not need authorization. We also have checked Fig 6 and deleted the genre retrieval to make sure they are all public paintings. For Fig 3 (new), we have confirmed that both paintings are from before 1924, which means these are in the public domain. All datasets we used are public datasets, as cited in the paper. We verified that we can use them without permission.

C4: We note that your manuscript is not formatted using one of PLOS ONE's accepted file types. Please reattach your manuscript as one of the following file types: .doc, .docx, .rtf, or .tex (accompanied by a .pdf). If your submission was prepared in LaTex, please submit your manuscript file in PDF format and attach your .tex file as "other".

R4: Our submission was prepared in LaTex and we have submitted our manuscript file in PDF format and attach .tex file as "other". We have pasted the contents of our .bbl into the appropriate position within the manuscript.

C5: Thank you for updating your figures to only include paintings that are currently in the public domain. Please be sure to update your figure captions to cite the paintings and state that they are in the public domain.

R5: By reviewing the submission guidelines and the articles that have been published, we have updated our figure captions to cite the paintings and state that they are in the public domain in Fig. 1, Fig. 3 and Fig. 6.

Special thanks to you for your good comments. We look forward to hearing from you regarding our submission. We would be glad to respond to any further questions and comments that you may have.

To Reviewer 1

This manuscript compared ResNet, ResNeXt, Res2Net, RegNetX, RegNetY and ResNeSt for art classification. The innovation of this work is limited. However, the comparison in this field is interesting. Some other problems in the manuscript are still concerned in the following:

C1: The organization of this manuscript should be added to the end of the introduction.

R1: We have added the organization of this manuscript to the end of the introduction. Thank you for the reminder.

C2: The flow charts of the compared models are suggested to be added.

R2: For the compared models, we have drawn flowcharts in Fig 2 to describe the different architectures of ResNet, Res2Net, ResNext and ResNest. In addition, we have drawn an overall general architecture in Fig 3 to better explain the overall structure. We use pseudocode to further explain our overall general architecture. It can be seen at the beginning of the Training Settings section. We designed a pipeline to draw a fair comparison between the models.

C3: More details on the compared models should be exposed in the text.

R3: We have revised the text according to the Reviewer's comments in the Convolutional Neural Network Models section. In order to better compare the role of the models' architecture in the classification of the art, the popular EfficientNet model has been for comparison in our experiments. We have tried our best to describe in detail the different structures of the 7 models. For the RegNet and EfficientNet models, we describe the basic network and

design process in detail. In addition, to better explain the different architectures of the models, we drew the blocks of the compared models in Fig 2.

C4: The results could be analyzed in details.

R4: We apologize for the lack of analysis, and we have rewritten this part according to the Reviewer's suggestion. The changes are described in detail below.

- We added a new comparison model and compared our work with previous work in Table 1. The results show that our results achieve state-of-the-art performance in all tasks on 3 datasets.
- We redrew the t-SNE visualization and Confusion matrix of style classification in Painting-91. We added the t-SNE visualization and Confusion matrix of genre classification in WikiArt. Combined with the Confusion matrix, we analyzed how the computer performs the art classification task. Furthermore, we explored how computers understand paintings.
- We performed a similarity search on the WikiArt dataset with 3 tasks instead of Painting-91 with 2 tasks. In other words, we expanded the image retrieval database. More similarity search results prove the validity of our model.

Special thanks to you for your good comments. We look forward to hearing from you regarding our submission. We would be glad to respond to any further questions and comments that you may have.

To Reviewer 2

After a careful review of this manuscript, I suggest my decision as accept after major revision. The following are some of my suggestions on this manuscript. I request the authors to make these required changes and resubmit the article after revisions.

C1: The title of the article is stated as "Comparing the performances of deep learning models on art classification tasks".But I find no proper system architecture in the proposed contribution. Adding an overall general architecture defining the entire system in the proposed section is much important.

R1: It is true, as the reviewer suggested, that it is appropriate to add an overall general architecture defining the entire system in the proposed section. We have built and drawn an overall general architecture in Fig 3 to make a fair comparison of the models in fine art classification. In addition, we use pseudocode to explain our overall general architecture. It can be seen at the beginning of the Training Settings section. In total, we design a pipeline to make a fair comparison between the models.

C2: Is there any particular reason to adopt DCNN algorithm?

R2: These are 3 reasons why we adopt the DCNN algorithm in our papers.

- Recently, an increasing number of papers have used convolutional neural network (CNN) models to solve classification problems. Many DCNN classification models with different architectures have been proposed and

achieve state-of-the-art performance on ImageNet. Our main goal is to explore the portability of different model structures in art classification. The results confirm that a model structure that performs better on ImageNet also performs better in the field of art.

- The establishment of large public datasets in the field of art makes it possible to use DCNN for classification. The most advanced algorithm for art classification in the public art collection is also currently obtained by DCNN, which can be seen in Table 1. Although DCNN is relatively uninterpretable, it has a good classification effect in the field of art. In the field of art, the interpretability of DCNN is also an important direction of our future research.

- QArt-learn has been introduced in the Introduction section. It uses a traditional machine learning method, such as KNN or SVM, to classify Baroque, Impressionism and Post-Impressionism styles. There are many tasks, and each task has numerous types. This makes it difficult to design features to distinguish between different classes. It is also why we use DCNN in our experiments.

C3: What can be potential advantage from a performance perspective?

R3: Through our experiments, we find that a simple model structure optimization can improve the classification accuracy in different art classification tasks. Compared with the previous work, our results achieve state-of-the-art performance in all classification tasks on different datasets. By using mixed precision training, our training speed has also been greatly improved. For better illustration, we do an experiment use resnet50 with 128 batchsize in MultitaskPainting100k, when use mixed precision, the speed is 509.26 images/s in the first epoch and the speed drops to 265.77 images/s when turn off mixed precision.

C4: Need more clarity on feature extraction process.

R4: In the Training Settings section, we build an overall general architecture defining the entire system that describes the working mechanism, including how to extract visual embeddings and how to use the embeddings in art classification tasks. In addition, we use pseudocode to further explain our overall process including feature extraction, update parameters of CNN and the use of visual embedding for classification.

C5: The overall significance of the work is not well-defined

R5: Our goal is to automatically classify and retrieve paintings in different attributes by computer. Our work helps the viewer better understand the paintings and explore how computers understand paintings. To better understand the meaning of this work, we highlight its overall significance in the Introduction section before the list of contributions.

C6: Results section needs much improvement.

R6: We apologize for the lack of analysis and have rewritten this part according to the reviewer's suggestion. The changes are described in detail below.

- We added a new comparison model and compared our work with previous work in Table 1. The results show that our results achieve state-of-the-art performance in all tasks on 3 datasets.

- We redrew the t-SNE visualization and Confusion matrix of style classification in Painting-91. We added the t-SNE visualization and Confusion matrix of genre classification in WikiArt. Combined with the Confusion matrix, we analyzed how the computer performs the art classification task. Furthermore, we explored how computers understand paintings.

- We performed a similarity search on the WikiArt dataset with 3 tasks instead of Painting-91 with 2 tasks. In other words, we expanded the image retrieval database. More similarity search results prove the validity of our model.

C7: The authors need to justify how the graphs defined in the result section contribute to the actual contribution of the work?

R7: T-SNE visualization and Confusion matrix graphs are used to explore how the computer performs the art classification task. We use t-SNE to reduce dimensionality and visualize the embedding distribution. We explore the computer classification mechanism through the confusion matrix and embedding distribution. In addition, we combine the actual meaning of the label and the contents depict in the painting to further explain the classification results displayed in the confusion matrix. More details can be seen in the Visualization and Discussion section, and we have rewritten most of this part.

C8: Adding more details on system configuration and types of tools used for simulation purpose along with appropriate specifications are more vital.

R8: It is really true as Reviewer suggested that adding more details on system configuration and types of tools used for simulation purpose along with appropriate specifications. We ran our experiments using Pytorch 1.5.0 in Ubuntu 18.04 with Titan RTX and Intel i9 10900k. All pretrained models we used are taken from code. These details are highlighted at the end of the Training Settings section.

C9: Add a real-world case study of the proposed scheme to understand the clarity of the work.

R9: We address the practical applicability of our results by analyzing different aspects of image similarity. We show that features derived from networks can be employed to retrieve images similar in either artist, genre or style, which can be used to enhance the capabilities of search systems on different online art collections. More details can be seen in the Similarity Search section. To better reflect the effort, we changed the retrieval source from Painting-91 to WikiArt and reviewed the images form the aspects of artist, style and genre. For copyright reasons, we show only the artist and style retrieval in the Fig 6.

C10: Organization of the paper require greater improvements.

R10: We apologize for our incorrect organization in the Results and Discussion section, where the Visualization and Discussion section and Similar Search section were subsections of the Results. We have corrected this and have tried our best to organize the structure of the article. We added the organization of the manuscript to the end of the Introduction. We deleted the Future work subsection from the Conclusion section to make the content more compact. We added the EfficientNet model, which has recently become popular, to bolster the content of the paper. We introduce

an overall general architecture in the Training Settings section, as described in comment 1. We have compared our work with previous work in Table 1. The results show that our results achieve state-of-the-art performance in all tasks on 3 datasets.

Special thanks to you for your good comments. We look forward to hearing from you regarding our submission. We would be glad to respond to any further questions and comments that you may have.

To Reviewer 3

Overall, the key source of the article is unique and excellent. In the whole process, the outcome should be well considered and recommend for acceptable with significant corrections

C1: There can be some resolutions problem with Fig.5 during publishing. If it is possible please re-draw them

R1: We have redrawn Fig 5, which appears as Fig 5 in the revised manuscript. In addition to the previous style classification in Painting-91, we added a confusion matrix of genre classification in WikiArt. Combined with the t-SNE visualization, we analyze how the models work in art classification tasks.

C2: Please comparing your work with other similar studies (related work)

R2: We have compared our work with previous work in Table 1. The results show that our results achieve state-of-the-art performance in all tasks on 3 datasets. Comparing different model architectures shows that in the case of little change in the numbers of model parameters, the model performance can be effectively improved by optimizing the model structure.

C3: The contribution and novelty should be better highlighted compared to the previous works

R3: Our contribution and novelty are in finding that the model architectures that perform well on ImageNet also perform well on the task of art classification, which has strong applicability, especially in larger art datasets. Previous works are compared using different tricks in Table 1 and more details are described in Results section. By simply optimizing the model structure, our results achieve state-of-the-art performance on all tasks. This has been highlighted in the Results section.

C4: There is no mention of the source code oused by the authors in this study (especially the deep learning framework used). This prevents the reproducibility of the study.

R4: We ran our experiments using Pytorch 1.5.0 in Ubuntu 18.04 with Titan RTX and Intel i9 10900k. All pretrained models we used are taken from code. These details are highlighted at the end of the Training Settings section.

C5: "Please bring strong relevance to the scope of journal using most recent two years" literature further to improve readership. Plos One is always the key word. Importantly, please look at most recent published articles from species and gender identification of mosquito. Please compare the findings with relevant studies to over-repeat the results and draw some constructive conclusions.

R5: After searching in Plos One, we found [1] [2], which achieves species and gender identification of adult mosquitoes using convolutional neural networks, are highly related to our work. We have added this reference in the

Transfer Learning section. In addition, we found [3] [4] [5], which are highly related to our work and are now cited it in our paper. We also compare our results with the previous work in Table 1, as stated in question 3.

C6: An old deep learning model (ResNet), please implement a new architecture.

R6: In order to better compare the role of the models' architectures in art classification, the popular EfficientNet model has been added as to our experiments a model for comparison. We have described the model in detail in the Convolutional Neural Network Models section, and the performance using Efficient is shown in Tables 2, 3, and 4. It shows this model has a good performance in art classification.

C7: The models are selected mentioning they have a smaller number of layers. This should not be the case. The optimal models need to be selected for the datasets under study through architecture and hyperparameter optimization.

R7: To make a fair comparison, we set the number of parameters in the models not differ widely to exclude the influence of the number of parameters on the classification results and focus on the model structure itself. A grid search of hyperparameters is time consuming and requires strong computing power. Thus, we performed a large number of experiments and selected a total of relatively good common hyperparameters. We focused on the architectures of models and froze other experimental conditions. By comparing different architectures in the art classification task, we found that the model architectures that perform well on ImageNet also perform well on the task of art classification. This finding has strong applicability, especially in larger art datasets.

Special thanks to you for your good comments. We look forward to hearing from you regarding our submission. We would be glad to respond to any further questions and comments that you may have.

Other changes:

- We modified the Transfer Learning section to better conform to the thesis theme and explained the role of transfer learning in our experiment.
- We added footnote in the Wikiart-WikiPaintings section to make it easier for the reader to view the image source.
- We have made some adjustments to the structure of the manuscript, revised some grammatical problems, corrected reference errors, combine some paragraphs and modified the figures.
- We unified the values in the tables to two decimal places.

References

[1] D. Motta, A. A. Bandeira Santos, I. Winkler, B. A. Souza Machado, D. A. Dias Imperial Pereira, A. M. Cavalcanti, E. O. Lins Fonseca, F. Kirchner, R. Badaro, Application of convolutional neural networks for classification of adult mosquitoes in the field, PLoS One 14 (1) (2019) e0210829. doi:10.1371/journal.pone.0210829.

[2] D. Motta, A. A. Bandeira Santos, B. A. Souza Machado, O. G. V. Ribeiro-Filho, L. O. Arriaga Camargo, M. A. Valdenegro-Toro, F. Kirchner, R. Badaro, Optimization of convolutional neural network hyperparameters for automatic classification of adult mosquitoes, Plos One 15 (7) (2020) e0234959. doi:10.1371/journal.pone.0234959.

[3] J. van Paasschen, F. Bacci, D. P. Melcher, The Influence of Art Expertise and Training on Emotion and Preference Ratings for Representational and Abstract Artworks, PLoS One 10 (8) (2015) e0134241. doi:10.1371/journal.pone.0134241.

[4] J. Cao, Y. Li, Y. Tian, Emotional modelling and classification of a large-scale collection of scene images in a cluster environment, PLoS One 13 (1) (2018) e0191064. doi:10.1371/journal.pone.0191064.

[5] B. Zafar, R. Ashraf, N. Ali, M. Ahmed, S. Jabber, S. A. Chatzichristofis, Image classification by addition of spatial information based on histograms of orthogonal vectors, PLoS One 13 (6) (2018) e0198175. doi:10.1371/journal.pone.0198175.
